# Supplementary material for: Orthogonal interlayer coupling in an all-antiferromagnetic junction
Source: Nat Commun. 2022 Jun 28;13:3723. doi: 10.1038/s41467-022-31531-w (PMC9240048; doi:10.1038/s41467-022-31531-w)
Supplement: Supplementary file 1 — Supplementary Information [file 41467_2022_31531_MOESM1_ESM.pdf]

Supplementary Materials for

Orthogonal interlayer coupling in an all-antiferromagnetic junction

Yongjian Zhou[1,3], Liyang Liao[1,3], Tingwen Guo[1], Hua Bai[1], Mingkun Zhao[2], Caihua Wan[2], Lin Huang[1], Lei Han[1], Leilei Qiao[1], Yunfeng You[1], Chong Chen[1], Ruyi Chen[1], Zhiyuan Zhou[1], Xiufeng Han[2], Feng Pan[1], Cheng Song[1✉]

[1]Key Laboratory of Advanced Materials (MOE), School of Materials Science and Engineering, Tsinghua University, Beijing 100084, China.

[2]Beijing National Laboratory for Condensed Matter Physics, Institute of Physics, University of Chinese Academy of Sciences, Chinese Academy of Sciences, Beijing 100190, China.

[3]These authors contributed equally to this work.

✉Corresponding author. E-mail: songcheng@mail.tsinghua.edu.cn

Note 1. Resistivity of samples

The resistivity measurements were carried out at room temperature. Corresponding results are shown in Fig. S1, confirming the highly insulating characteristic of samples.

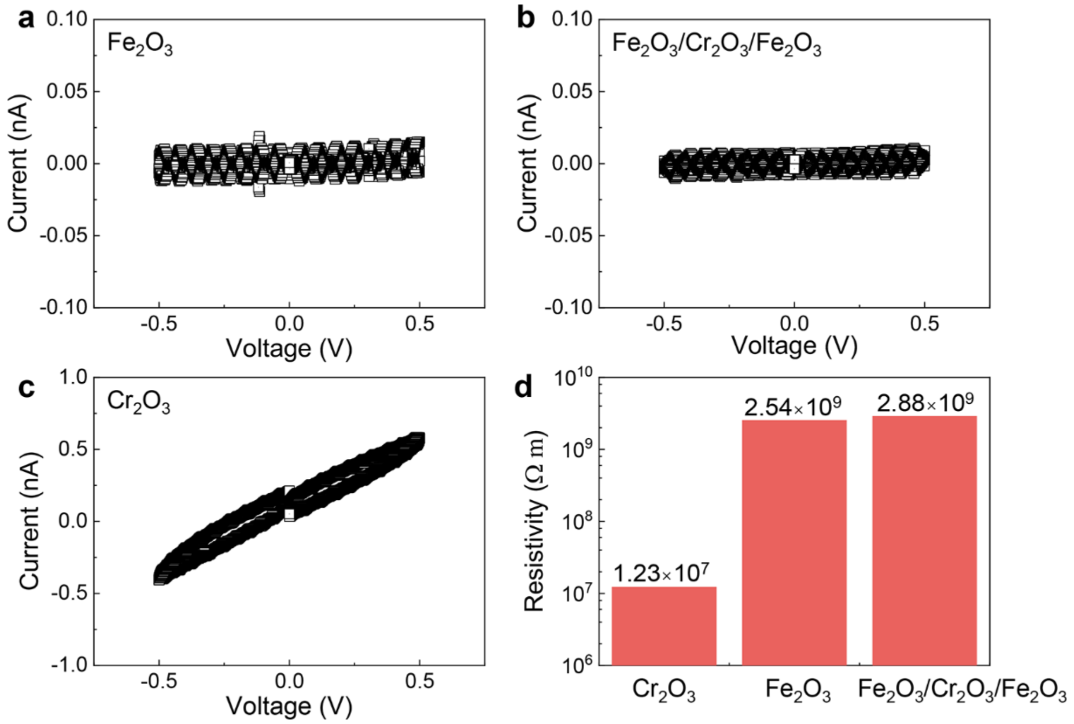

Fig. S1 Resistivity of samples. Current-voltage (*I*-*V*) curves of Fe_2O_3 (**a**), $Fe_2O_3/Cr_2O_3/Fe_2O_3$ (**b**) and Cr_2O_3 (**c**). These films were sandwiched by two electrodes for the *I*-*V* measurements. Corresponding resistivity data are calculated and shown in **d**. The ultralow current and concomitantly high resistivity reflect the high insulating characteristic (>GΩ) of the samples. Note that the values of current in **a** and **b** are at the order of pA, which is close to the limit of the sensitivity of semiconductor device analyzer, therefore there exists some noise.

Note 2. Simulation of the SMR curves

SMR curves of the control samples (Fig. 2a and b in the main text) show hysteretic behavior, suggesting that the Néel vector is switched when the Zeeman

energy overcomes the anisotropy energy. The switching reflects that the degeneracy of the spin-flop states is broken, which is caused by the Dzyaloshinskii-Moriya interaction (DMI)[S1]. Meanwhile, the curves have no sharp resistance transition, revealing that the switching is step-by-step, because the anisotropy scatters in a certain scale. To mimic these behaviors, we calculate the energy profile of different magnetic configurations to obtain the transition conditions under a given anisotropy. The SMR curves are simulated by varying the anisotropy and estimating the sum of the curves with corresponding transition conditions.

The energy of one Fe_2O_3 layer can be expressed as

$$E_1 = -M_S\mathbf{m}_1\cdot\mathbf{H} - M_S\mathbf{m}_2\cdot\mathbf{H} + \frac{1}{2}M_S H_E\mathbf{m}_1\cdot\mathbf{m}_2 + M_S H_{DM}\mathbf{m}_1\times\mathbf{m}_2 + K(\mathbf{m}_1) + K(\mathbf{m}_2)\,, \tag{S1}$$

where $\mathbf{H}$ is the external magnetic field, $\mathbf{m}_i$ (i = 1, 2) is the direction vector of the magnetization of sublattice i, M_S is the saturated magnetization of one sublattice, H_E is the exchange field, H_{DM} is the DMI effective field and $K(\mathbf{m}_i)$ is the anisotropy. Due to the strong exchange coupling[S2,S3], we have $\mathbf{m}_1 \approx -\mathbf{m}_2$, thus it is convenient to describe the magnetic configuration by the Néel vector $\mathbf{m}_1 - \mathbf{m}_2$ and the angle α between $\mathbf{m}_1$ and $-\mathbf{m}_2$ ($\alpha \ll 1$). Set the external field at x-axis and minimize the energy by varying α, we have

$$E = -M_S\frac{(H\sin\theta + H_{DM})^2}{H_E} + 2M_S H_A\cos^2(3\theta) \quad \text{(S2)}$$

where θ is the angle between the Néel vector and the x-axis, and H_A is the three-fold anisotropy effective field[S4,S5]. Note that the influence of α on the anisotropy energy is neglected because the anisotropy field is much smaller than the exchange field, the external field and the DMI effective field[S2,S3,S6].

We then calculate the energy profile of $\theta = \pi/2$, π and $3\pi/2$ states. The switching condition between these states are obtained by solving $E(\theta_1) = E(\theta_2) + 4K_3$, where θ_1 and θ_2 are the states before and after the switching, respectively. The $4K_3$ term is added to mimic the barrier between two states. The normalized SMR signal is equal to $\cos^2\theta$, therefore, when the system goes through the $\theta = \pi/2$, π and $3\pi/2$ states, the signal would be 0, 1 and 0, respectively.

As mentioned above, the anisotropy of the sample has a distribution, and the exact form of this distribution is unknown. However, the peak position and the peak width could reflect the center and the range of the distribution of the anisotropy. To give a simple and reasonable approximation, we assume that the distribution of the anisotropy results in a normal distribution $N(\mu, \sigma^2)$ of the switching condition, where μ and σ can be estimated by the average and the standard deviation of the switching conditions calculated from different anisotropy parameters, respectively.

We calculated two SMR curves with $H_A = \{1, 3, 5\}$ and $\{0.4, 1.2, 2\}$ (in Oe), shown in Fig. S2a and b, respectively. Other parameters are set to be $H_E = 9 \times 10^6$ Oe and $H_{DM} = 2 \times 10^4$ Oe[S2,S3,S6]. It can be seen that the simulated curves of the first and second H_A groups are in good agreement with the experimental curves of the Fe_2O_3 and Cr_2O_3/Fe_2O_3 control samples, respectively.

The SMR curve in the antiferromagnetic junction with interlayer coupling (Fig. 2d in the main text) is obtained in a similar way. The energy of the system is given by

$$E = -t_b M_S \frac{(H\sin\theta_b + H_{DM})^2}{H_E} - t_t M_S \frac{(H\sin\theta_t + H_{DM})^2}{H_E} + 2t_b M_S H_{Ab}\cos^2(3\theta_b) + 2t_t M_S H_{At}\cos^2(3\theta_t) + J\sin^2(\theta_t - \theta_b), \tag{S3}$$

where t_b and t_t are the thickness of the bottom and top layers, respectively, H_{Ab} and H_{At} are the anisotropy field of the bottom and top layers, respectively, θ_b and θ_t are the

θ of the bottom and top layers, respectively, and J is the interlayer coupling energy. (θ_b, θ_t) = (π/2, π/2), (π/2, π), (π, 3π/2), (3π/2, 0) and (3π/2, 3π/2) are considered, corresponding to normalized SMR signal 0, 1, 0, 1 and 0, respectively. In SMR curve calculation, when both H_{Ab} and H_{At} are involved in the switching condition, we estimate the σ parameter in the normal distribution $N(\mu, \sigma^2)$ by the sum of the standard derivation of the switching condition caused by each anisotropy field. Using H_{Ab} = {1, 3, 5} and H_{At} ={0.4, 1.2, 2} (in Oe), $J/M_S t_b$ = 7 Oe (corresponding to coupling energy at the order 0.01 meV/unit cell), t_b = 12 nm, t_t = 4 nm, we obtain the calculated SMR curve of the antiferromagnetic junction as shown in Fig. 2e in the main text. The calculated curve reproduces the key features of the experimental curve displayed in Fig. 2d in the main text, demonstrating the existence of an orthogonal interlayer coupling in the antiferromagnetic junction.

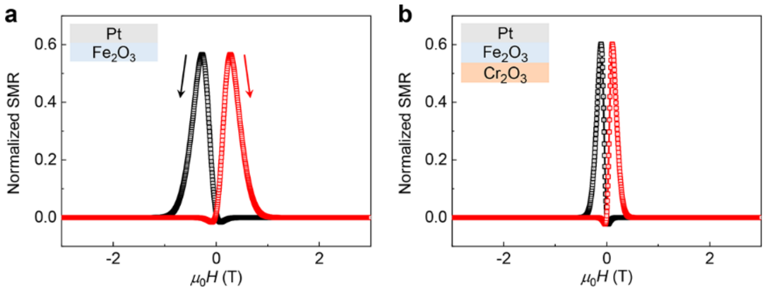

Fig. S2. Simulated SMR curves in control samples. a, Fe_2O_3/Pt **b**, Cr_2O_3/Fe_2O_3/Pt. The resistance peak in Fe_2O_3/Pt appears at larger magnetic field than that of Cr_2O_3/Fe_2O_3/Pt, indicating a larger spin-flop field in Fe_2O_3/Pt, which is consistent with the experimental results in Fig. 2A,B in the main text.

Note 3. HAADF-STEM image of Fe_2O_3/Cr_2O_3/Fe_2O_3

Figure S3a presents a high-angle annular dark-field scanning transmission electron microscopy (HAADF-STEM) image of the Fe_2O_3/Cr_2O_3/Fe_2O_3 cross-section.

The Fourier transform along the $[\bar{1}\bar{1}20]$ zone axis of Cr_2O_3 (Fig. 3b) and the bottom Fe_2O_3 (Fig. 3c) reflects the epitaxial growth of the junction.

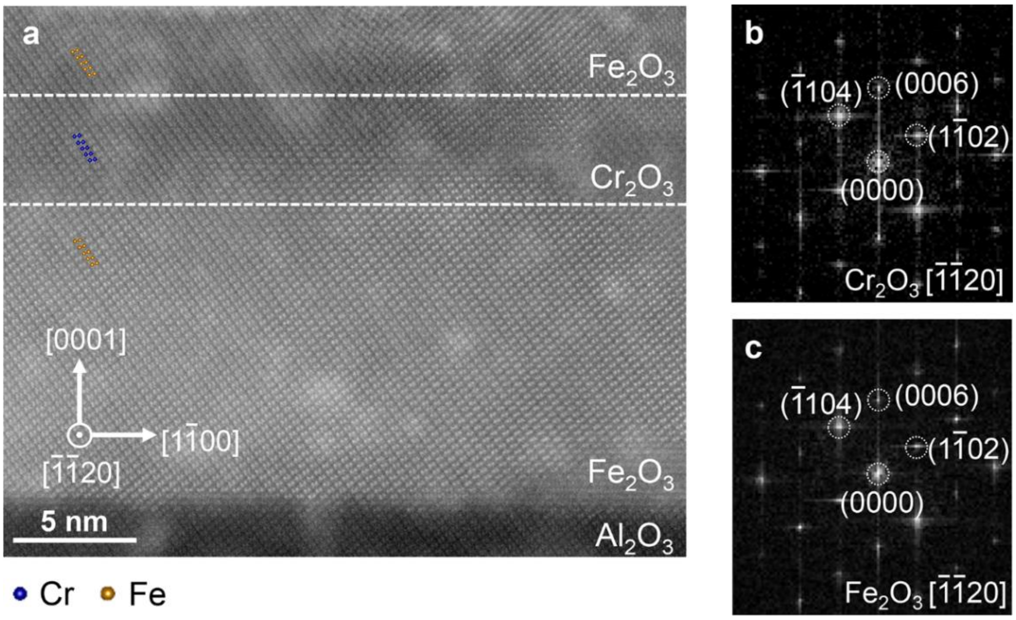

Fig. S3. HAADF-STEM image and Fourier transform patterns. a, HAADF-STEM image of the $Fe_2O_3/Cr_2O_3/Fe_2O_3$ junction along the $[\bar{1}\bar{1}20]$ zone axis, where the white dashed lines denote the interfaces. Cr and Fe atoms are highlighted. Note that there exist some bright islands, which is caused by adsorbed platinum during sample preparation by focused ion beam (FIB). The Fourier transform along the $[\bar{1}\bar{1}20]$ zone axis of Cr_2O_3 (**b**) and the bottom Fe_2O_3 (**c**) reflects the epitaxial growth.

<u>Note 4. Magnetoresistance measurement with low spin Hall material as the cap layer.</u>

In order to verify that the magnetoresistance is caused by SMR, we performed measurement with the cap layer comprised by a low spin Hall material titanium (Ti) under the same test condition with that in $Fe_2O_3/Cr_2O_3/Fe_2O_3$/Pt. The corresponding data are shown in Fig. S4. It can be seen that there exists negligible magnetoresistance

signal when the magnetic field is applied, excluding other magnetoresistive effect such as anisotropic magnetoresistance.

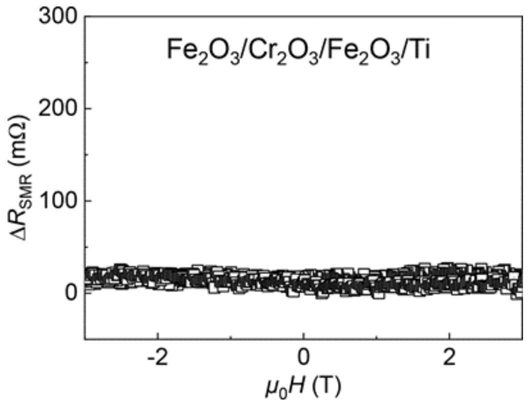

Fig. S4. Negligible magnetoresistance in $Fe_2O_3/Cr_2O_3/Fe_2O_3/Ti$.

Note 5. Exclusion of the influence from defects-related magnetism

In antiferromagnets, the SMR shows a negative polarity, namely, low resistance states at high H reflect that the Néel vector (n) of Fe_2O_3 is perpendicular to the current (x) due to the spin-flop at high fields and deviates towards the current direction at low fields[S8–S10]. In ferromagnets, it is well established that the sample exhibits a low resistance state at low H (at the coercivity). Therefore, to exclude the effect from weak magnetism (caused by defects or uncompensated interface), we measured the magnetic hysteresis loop (M-H) of the sample and show the data in Fig. S5a. Concomitant dM/dH is calculated to characterize the coercivity of the remanent magnetism. The SMR and dM/dH curves of $Fe_2O_3/Cr_2O_3/Fe_2O_3/Pt$ at 300 K are presented together in Fig. S5b. To compare the magnetic field corresponding to the valley of SMR and the coercivity, an enlarged region between –0.7 and 0.7 T is illustrated in Fig. S5c, where the ranges of the SMR valley and coercivity are marked by the dotted lines (purple region) and solid lines (grey region), respectively. Remarkably, the two regions are located at completely different magnetic fields,

indicating that they have no connection. Thus the valley in the SMR curves is not caused by the defects-related magnetism, and any possible coupling between net moment in Cr_2O_3 and *n* in Fe_2O_3 are excluded.

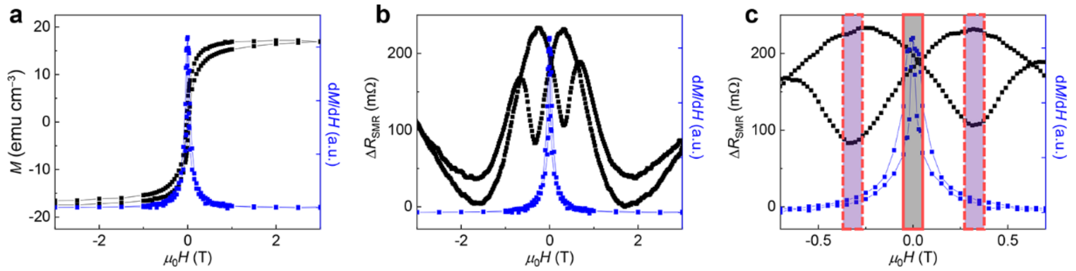

Fig. S5. Exclusion of the influence from defects-related magnetism. The *M-H* loop and derivative of the *M-H* loop (**a**) and SMR (**b**) of the $Fe_2O_3/Cr_2O_3/Fe_2O_3$/Pt sample at 300 K. An enlarged region between –0.7 and 0.7 T is illustrated in **c**, where the ranges of the SMR valley and coercivity are marked by the dotted lines (purple region) and solid lines (grey region), respectively.

Note 6. SMR in Cr_2O_3/Fe_2O_3/Pt at various magnetic fields and temperatures

Figure. S6 shows the angle dependence of SMR in Cr_2O_3/Fe_2O_3/Pt under various magnetic field and temperature. In the control samples, at all of the temperatures and magnetic fields used here, the polarities of angle dependent SMR are always negative, reflecting the stable antiferromagnetic feature of Fe_2O_3 and the Néel vector is perpendicular to the magnetic field (spin-flop state) because of the absence of interlayer coupling effect. The magnetic field dependence of SMR (Fig. S6c) also shows the characteristic of antiferromagnetic SMR. Note that the Cr_2O_3 grown on Al_2O_3 substrate should possess Néel temperature within this temperature range[S7]. Therefore, the net moment in Cr_2O_3 above the Néel temperature cannot lead to interlayer coupling. The angle and field dependence of SMR further supports the existence of interlayer coupling in our $Fe_2O_3/Cr_2O_3/Fe_2O_3$ antiferromagnetic junction.

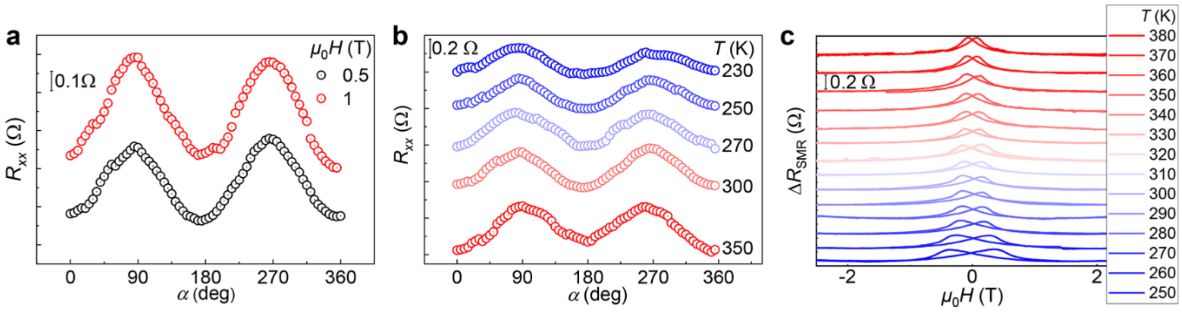

Fig. S6. SMR in Cr_2O_3/Fe_2O_3/Pt at various magnetic fields and temperatures. a, SMR under various magnetic fields μ_0H = 0.5 and 1 T. **b,** Angle dependence of SMR at different temperatures with magnetic field μ_0H = 0.5 T. **c**, Magnetic field dependence of SMR at different temperatures. The curves are shifted vertically for clarity.

Note 7. Angle dependence of SMR in Fe_2O_3/Pt and Cr_2O_3/Fe_2O_3/Pt

The angle dependence of SMR in Fe_2O_3/Pt and Cr_2O_3/Fe_2O_3/Pt is shown in Fig. S7. As the magnetic field increases to around 0.2 T, the SMR curve in Cr_2O_3/Fe_2O_3/Pt exhibits negative polarity, namely, the low resistance state is obtained when $n \perp I$, which is the characteristic of antiferromagnetic SMR[S8–S10], indicating the Néel vector in the top Fe_2O_3 tends to be aligned in spin-flop state ($n \perp H$) gradually. However, the SMR curve in Fe_2O_3/Pt presents a negative polarity until the magnetic field reaches 0.35 T. The results indicate that the spin-flop field (marked with solid symbols) is higher for the bottom Fe_2O_3 (~0.35 T) than that of the top Fe_2O_3 (~0.2 T).

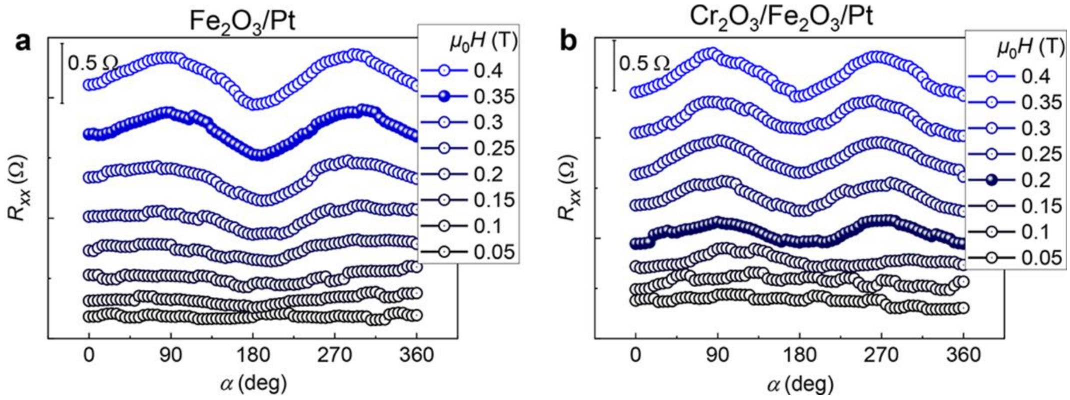

Fig. S7. Angle dependence of SMR. Angle dependence of SMR in Fe_2O_3(12)/Pt(4) (**a**) and Cr_2O_3(4.4)/Fe_2O_3(4)/Pt(4)(units in nanometers) (**b**) for different magnetic fields as marked at 300 K.

Note 8. SMR in Fe_2O_3(4)/Cr_2O_3(4.4)/Fe_2O_3(12)/Pt

The SMR in sandwich with inverted structure, Fe_2O_3(4)/Cr_2O_3(4.4)/Fe_2O_3(12), is performed to detect the direction of Néel vector in thicker Fe_2O_3 and shown in Fig. S8. The resistance peaks appear after $\mu_0 H$ = 0 (approximately $\mu_0 H$ = ±0.3 T), demonstrating that the Néel vector in the 12 nm-thick Fe_2O_3 maintains spin-flop state owing to the larger Zeeman energy rather than deviating towards H at low magnetic field. It indicates that the top and bottom Fe_2O_3 in main text should be orthogonally coupled. In addition, the spin-flop field of 12 nm-thick Fe_2O_3 grown on Cr_2O_3 is larger than that of 4 nm Fe_2O_3.

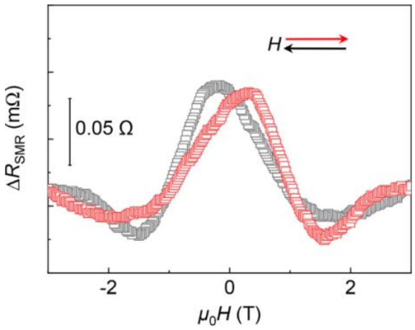

Fig. S8. SMR in Fe_2O_3(4)/Cr_2O_3(4.4)/Fe_2O_3(12)/Pt.

Note 9. XMLD spectra and corresponding antiferromagnetic structures

Fe L-edge x-ray magnetic linear dichroism (XMLD) was also conducted to further explore the coupling effect in antiferromagnets (AFM). Fig. S9a–c shows the XMLD spectra (left panel, also shown in Fig. 2f–h in the main text) and AFM Néel order (right panel) for Fe_2O_3/Cr_2O_3/Fe_2O_3, Fe_2O_3 and Cr_2O_3/Fe_2O_3, respectively. The orange

arrows in right panel represent Néel vectors along three easy-axes in the top Fe_2O_3. A high magnetic field was pre-applied along one of the axes (black arrow, *x*-axis), then removed to perform XMLD, benefiting from the non-volatile characteristic of Fe_2O_3[S6]. X-ray was vertically incident to the film and the polarized direction of the x-ray was parallel to the film plane (Fig. S9d). The chamfered edge here is $[11\bar{2}0]$, the direction perpendicular to which is one of the easy-axes and is also the direction of applied field. The XMLD spectra (black line) were taken from the differences of XAS spectra ($XAS_{\perp} - XAS_{//}$) and then multiply by a factor of 5 at the absorption edges for clarity. The XMLD spectrum in Fig. S9a shows a zero–positive–negative–zero feature, indicating that the Néel vector is mainly along the parallel direction (*n* // *x*-axis), represented by bold orange arrow (right panel in Fig. S9a), confirming the existences of interlayer coupling. In Fe_2O_3 and Cr_2O_3/Fe_2O_3, the XMLD spectra present zero–negative–positive–zero feature, suggesting that the Néel vectors in Fe_2O_3 are mainly aligned along the spin-flop direction (*n* // *y*-axis) without interlayer coupling (bold orange arrows in Fig. S9b,c right panel). Considering the three-fold easy-axes of Fe_2O_3, the Néel vectors in the control samples should be aligned along the other two easy-axes with the main components along the direction perpendicular to the magnetic field rather than along it[S6].

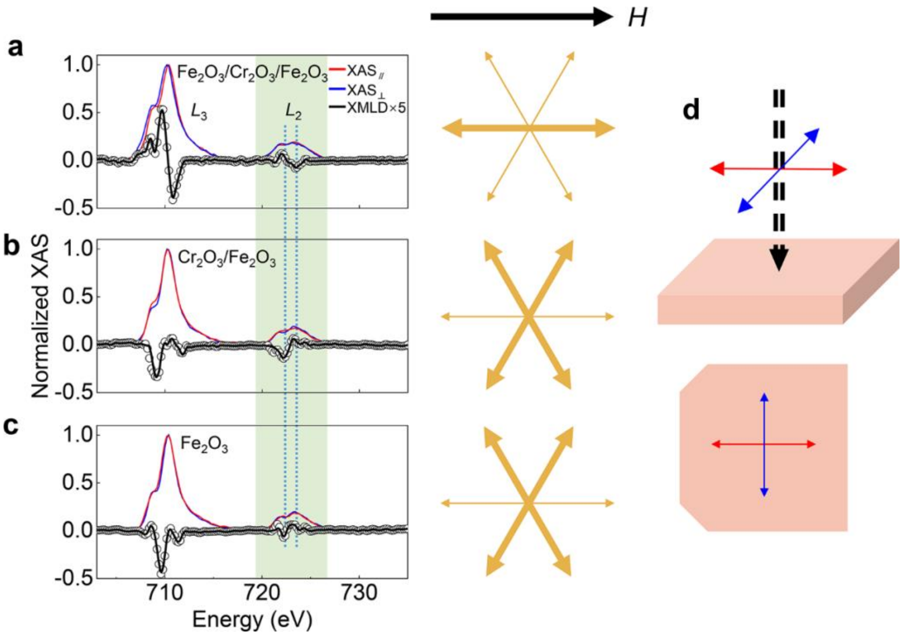

Fig. S9. XMLD spectra and experiment set-ups. a–c, Normalized XAS spectra, XMLD results and corresponding schematic diagram of the magnetic structures in $Fe_2O_3/Cr_2O_3/Fe_2O_3$ (**a**), Cr_2O_3/Fe_2O_3 (**b**) and Fe_2O_3 (**c**). The highlighted region denotes Fe-L_2 edge and the vertical dotted lines are guide for eyes to mark the valley and peak in XMLD curves. The orange arrows denote the Néel vectors in the top Fe_2O_3. **d,** Schematic illustration of XAS experiments. The x-ray was vertically incident to the film with polarized direction (red and blue arrows) shown in the lower panel.

The XMLD measurements with $Fe_2O_3/Cr_2O_3/Fe_2O_3$ rotation in the film plane were performed to further determine the interlayer coupling and the direction of the Néel vector in the top Fe_2O_3. The XMLD spectra at 0°, 30° and 60° are presented in Fig. S10a–c, and the corresponding XAS polarization set-ups as well as Néel order structures are shown in Fig. S10d–f, respectively. When $XAS_{//}$ is parallel to the magnetic field direction, the Néel vector along $XAS_{//}$ is larger than that along $XAS_{\perp}$ due to the interlayer coupling effect as discussed above and in the main text. When

the sample is rotated by 30°, the component of Néel vector along $XAS_{//}$ (red line in Fig. S10e right panel) is slightly larger than that along $XAS_{\perp}$ (blue line in Fig. S10e right panel), resulting in a relatively weak XMLD signal in Fig. S10b. And the polarity of XMLD is still the same as that at 0° (Fig. S10a). However, when the sample is rotated by 60°, the polarity of XMLD reverse, namely, the component of Néel vector along $XAS_{//}$ is smaller than that along $XAS_{\perp}$[S11,S12], as shown by red and blue lines in Fig. S10f. Through the analysis above, the Néel vector of the top Fe_2O_3 is demonstrated to be mainly aligned parallel with the direction of applied magnetic field ($XAS_{//}$), confirming the existence of interlayer coupling in AFM.

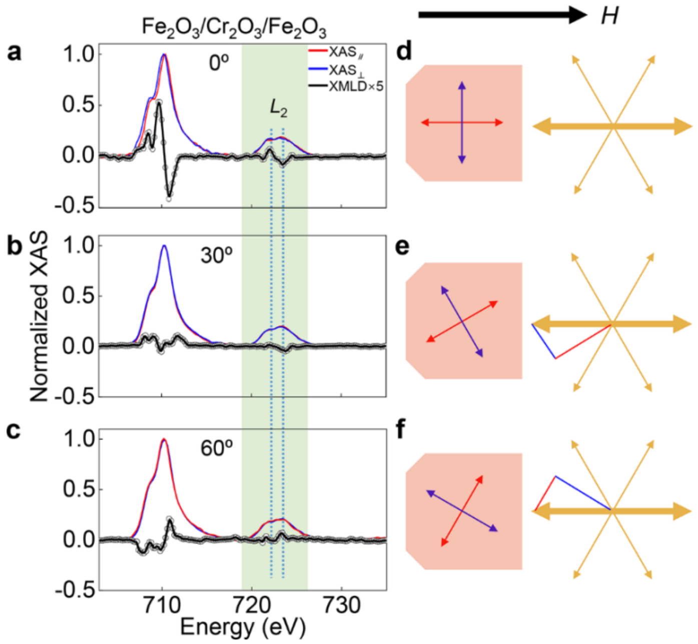

Fig. S10. XMLD spectra for different in-plane rotation angles. a–c, XAS and XMLD spectra of $Fe_2O_3/Cr_2O_3/Fe_2O_3$ for different various in-plane rotation angles, 0° (**a**) 30° (**b**) and 60° (**c**). **d–f,** Schematic diagram of the experimental set-ups and magnetic structures for different in-plane rotation angles, 0° (**d**) 30° (**e**) and 60° (**f**). The polarized directions are shown by red and blue arrows in (**d–f**)**.** The orange arrows represent the Néel vectors in the top Fe_2O_3. The red and blue lines denote the component of Néel vectors along $XAS_{//}$ and $XAS_{\perp}$, respectively.

Note 10. SMR in $Fe_2O_3/Cr_2O_3/Fe_2O_3$/Pt at various temperatures

The magnetic field dependence of SMR in $Fe_2O_3/Cr_2O_3/Fe_2O_3$/Pt as a function of temperature are shown in Fig. S11. As the temperature decreases, the second resistance peak decays gradually and the location of first resistance peak shifts towards H = 0. At very low temperatures, such as 100 K, the resistance peak appears almost at zero-field. This feature is most likely due to the lower spin-flop field of Fe_2O_3 resulting from the decrease of the in-plane anisotropy.

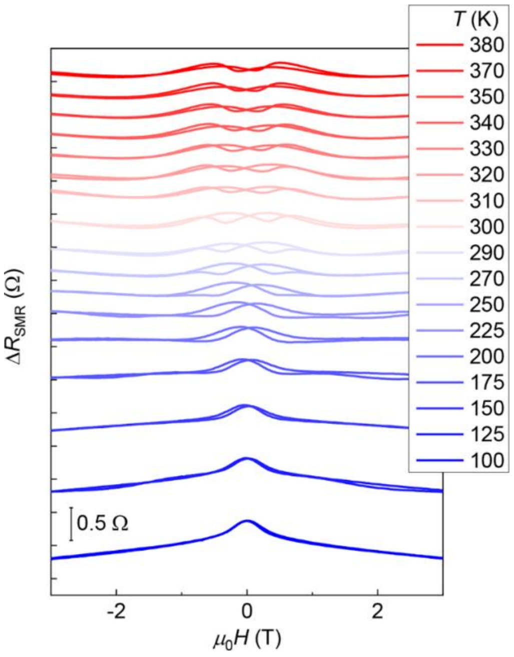

Fig. S11. Magnetic field dependence of SMR in $Fe_2O_3/Cr_2O_3/Fe_2O_3$/Pt at various temperatures. The curves are shifted vertically for clarity.

Note 11. SMR signals of $Fe_2O_3/Cr_2O_3/Fe_2O_3$/Pt with magnetic field applied along different directions

The measurement of SMR with magnetic field applied along different directions are performed with results shown in Fig. S12. In the two cases, the SMR results have very similar characteristics, especially the magnetic fields corresponding to the first

resistance peak are almost the same. This observation discloses that the interlayer coupling always exists, irrespective of the magnetic field direction.

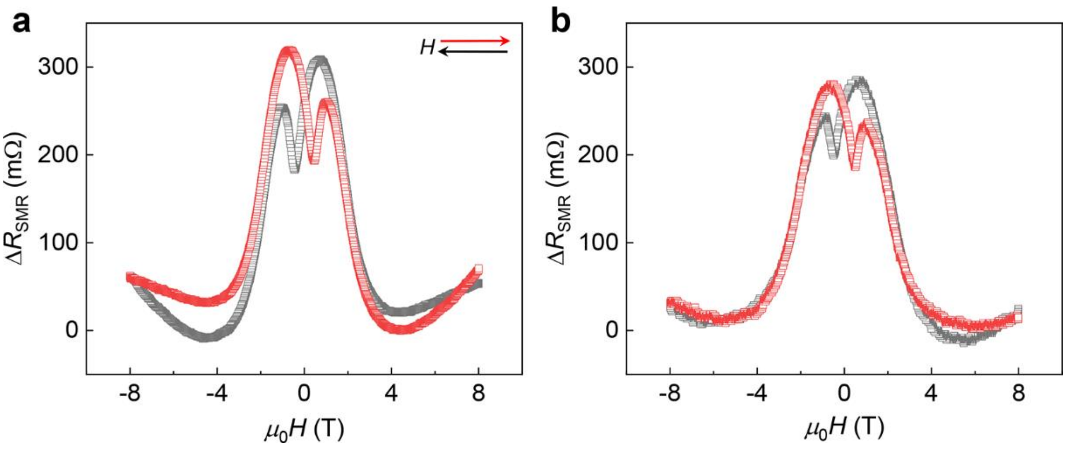

Fig. S12. SMR signals of $Fe_2O_3/Cr_2O_3/Fe_2O_3$/Pt with magnetic field applied along different directions. a, parallel with one of the easy-axes **b,** perpendicular to one of the easy-axes.

<u>Note 12. SMR in $Fe_2O_3/Cr_2O_3/Fe_2O_3$/Pt with different reading current</u>

We have measured the field dependence of SMR with current of different signs and magnitudes as shown in Figure. S13. The field dependence of SMR curves varied little with current, indicating the interlayer coupling is an intrinsic property of the structure. The results exclude thermal artifacts such as Seebeck effect or Nernst effect or unidirectional SMR where ΔR is proportional to I.

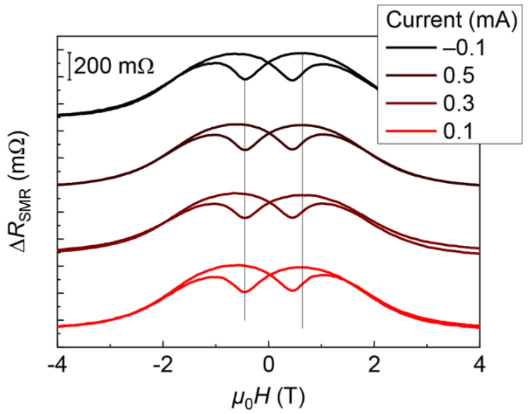

Fig. S13. Field dependence of SMR measured with different currents. The applied current has no remarkable influence on the two resistance peak of SMR, indicating interlayer coupling is an intrinsic effect instead of thermal artifacts.

Note 13. Thickness variation and interfacial roughness in Cr_2O_3.

The interfacial roughness in Cr_2O_3 layer is important in our theoretical model. Therefore, the thickness variation and interfacial roughness are measured via atomic force microscope, and the corresponding data are shown in Fig. S14. It can be seen that the variation of Cr_2O_3 thickness is at the order of several angstroms (Fig. S14a). To further support the thickness variation, the roughness data along two diagonal lines are shown in Fig. S14b and c. The thickness variation is large enough (the order of several angstroms) for the co-existence of parallel- and antiparallel-preferred areas in Fe_2O_3 layers[S13], further supporting our theory model.

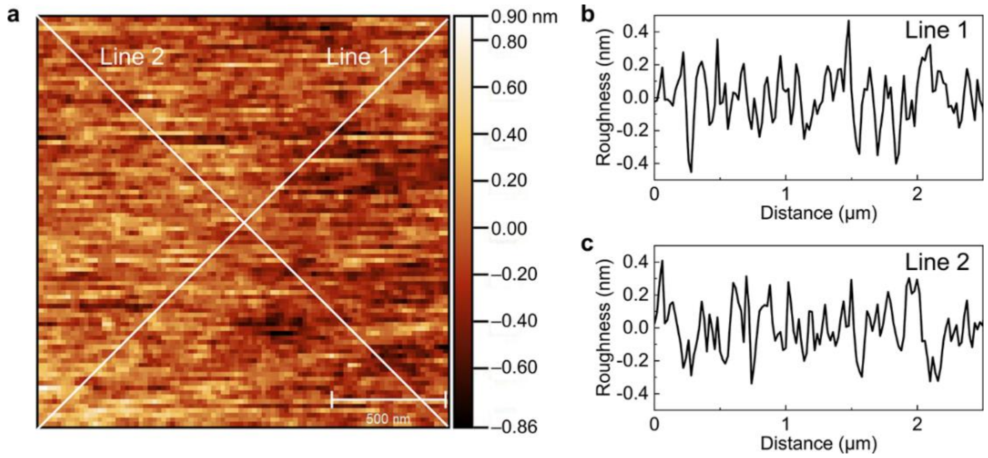

Fig. S14 Thickness variation and interfacial roughness in Cr_2O_3. a, atomic force microscope data. **b, c,** the roughness data along two diagonal lines.

Note 14. Maximum coupling field as a function of Cr_2O_3 thickness

To further investigate the coupling effect and the spin structure in Cr_2O_3, the maximum coupling field ($\mu_0 H_{MaxCoupling}$) as a function of Cr_2O_3 thickness is plotted.

The maximum coupling field is inversely proportional to the square of the Cr_2O_3 thickness t, (Fig. S15) which is consistent with our model based on the non-uniform domain wall state (Eq. 3 in the main text). Such a consistency verifies the proposed spin structure in Cr_2O_3 in the non-uniform domain wall model.

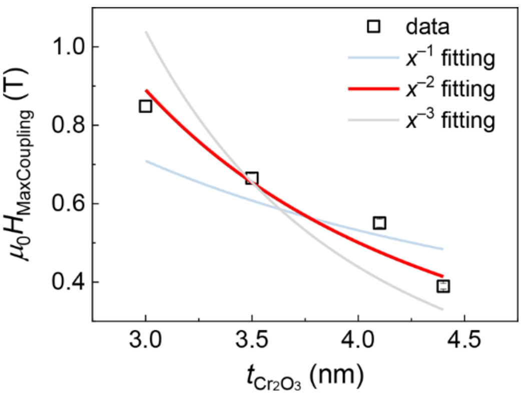

Fig. S15 Maximum coupling field ($\mu_0 H_{MaxCoupling}$) as a function of the square of the Cr_2O_3 thickness t. The maximum coupling field is inversely proportional to the square of t.

Note 15. Magnetic field dependence of SMR in $Fe_2O_3/Cr_2O_3/Fe_2O_3$/Pt with various Cr_2O_3 thicknesses

The magnetic field dependence of SMR results with different thickness of Cr_2O_3 are shown in Fig.S16. Note that the summarized data in Fig. 4a,b in the main text are the average values of these data and their counterparts with the opposite sweeping. There are two most eminent features in this figure: (i) The location ($\mu_0 H_{Coupling}$ in the main text) of the first peak varies with temperatures, proving that the coupling effect in AFM is temperature-dependent; (ii) the second resistance peak (MR signal in the main text) at the negative magnetic field drops gradually and vanishes eventually with decreasing temperatures. Moreover, the coupling effect still exists even at the temperatures where the MR signal disappears.

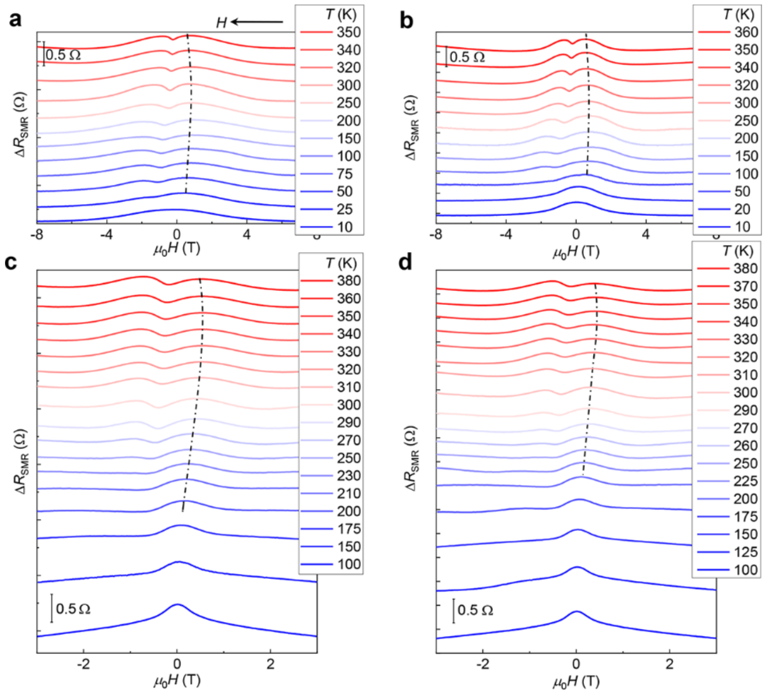

Fig. S16. Magnetic field dependence of SMR in $Fe_2O_3/Cr_2O_3/Fe_2O_3$/Pt with various Cr_2O_3 thicknesses. **a,** 3.0 nm, **b,** 3.5 nm, **c,** 4.1 nm, and **d,** 4.4 nm. For clarity, only the magnetic field sweeping along one direction (depicted by black arrow, from positive to negative) is displayed. The dashed lines are guide for eyes. The curves are shifted vertically for clarity.

Note 16. Absence of interlayer coupling with thicker Cr_2O_3 spacer

As the thickness of Cr_2O_3 spacer increases to t = 6 nm, only one resistance peak exists at negative magnetic field in Fig. S17a when sweeping H from positive to negative, indicating the absence of the interlayer coupling. A similar behavior is also observed when t = 12 nm (Fig. S17b). This feature can be explained as follows: in thicker Cr_2O_3 spacer, the magnetic structure maintains ground state rather than entering the NUDW state. Thus no coupling exists.

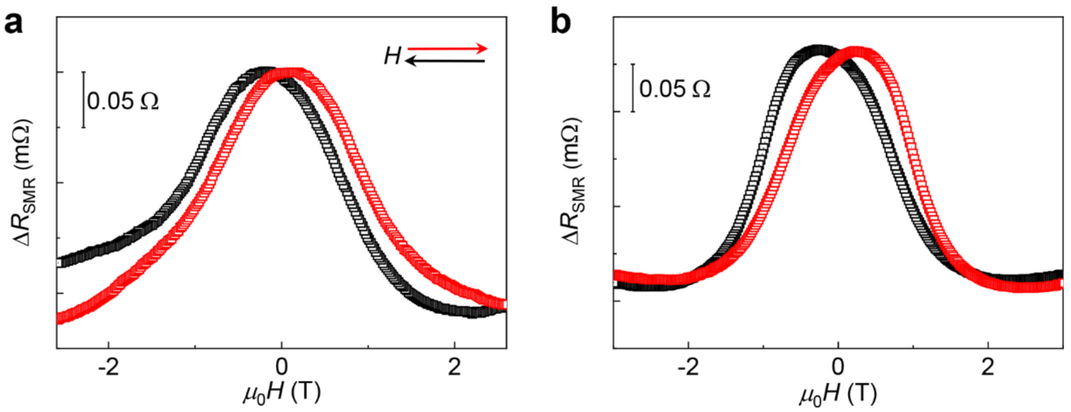

Fig. S17. Magnetic field dependence of SMR in the $Fe_2O_3/Cr_2O_3(t)/$ Fe_2O_3/Pt samples at 300 K for comparatively thick spacer. t = 6 nm (**a**) and t = 12 nm (**b**).

Note 17. Interlayer coupling in antiferromagnet with other spacer layer

Apart from the Cr_2O_3 spacer, we also used nickel oxide NiO as the spacer layer to prepare $Fe_2O_3(15)/NiO(5)/Fe_2O_3(15)$ (units in nanometers) junctions, which was deposited by radio frequency sputtering. Identical SMR measurements were carried out in the $Fe_2O_3/NiO/Fe_2O_3/Pt$ samples. There exist two resistance peaks in the SMR curves (T = 250 K) in Fig. S18a, one at positive H and the other at negative H, similar to the $Fe_2O_3/Cr_2O_3/Fe_2O_3/Pt$ case. This features as the interlayer coupling between the two Fe_2O_3 layers. Also visible is the two peaks persistently exist when decreasing the temperature to 125 K (Fig. S18b). It is then concluded that the interlayer coupling effect is not restricted to a certain spaced material.

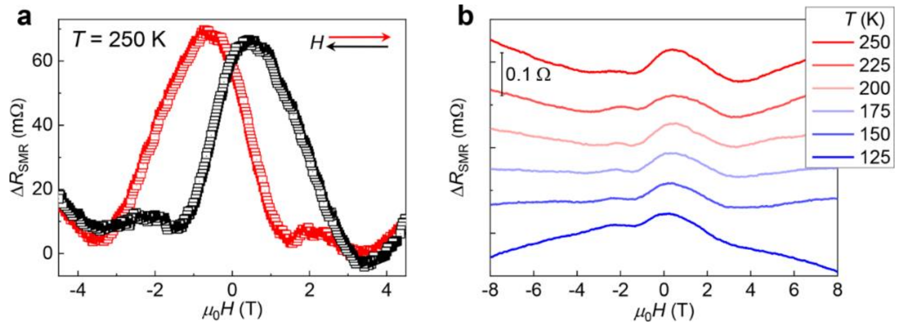

Fig. S18. Magnetic field dependence of SMR in Fe_2O_3/NiO/Fe_2O_3/Pt. a, Magnetic field dependence of SMR at T = 250 K. The arrows denote the sweeping direction of magnetic field. **b,** Magnetic field dependence of SMR at different temperatures with H sweeping from positive to negative.

In addition, the SMR measurements were carried out in $Fe_2O_3/Al_2O_3/Fe_2O_3$/Pt samples with a simple non-magnetic spacer (4 nm-thick Al_2O_3). Corresponding data are shown in Fig. S19. Only one resistance peak appears at $H < 0$ ($H > 0$) with H sweeping from positive to negative (negative to positive), which is quite characteristic for the negative SMR of the top antiferromagnetic Fe_2O_3 layer. And this feature is similar to the SMR data of Fe_2O_3 (Fig. 2a) and Cr_2O_3/Fe_2O_3 (Fig. 2b), reflecting the absence of interlayer coupling between the Fe_2O_3 layers when the spacer is Al_2O_3. This observation also supports that the non-uniform domain wall state in Cr_2O_3 spacer play a fundamental role on the interlayer coupling between Fe_2O_3 layers.

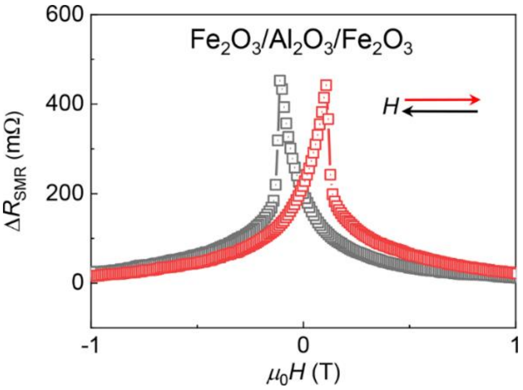

Fig. S19. Magnetic field dependence of SMR in $Fe_2O_3/Al_2O_3/Fe_2O_3$/Pt. The arrows denote the sweeping direction of magnetic field. There exists only one resistance peak at $H < 0$ ($H > 0$) with H sweeping from positive to negative (negative to positive).

<u>Note 18. Coupling energy calculation</u>

The temperature dependence of the free energy under the perpendicular and the parallel conditions, $F(\Delta_{//},T)$ and $F(\Delta_{\perp},T)$, can be obtained by solving (1)(2) with $\Delta_{\perp}$ and $\Delta_{//}$, respectively. The coupling energy can be calculated by taking the free energy difference under the two conditions

$$E_C = F(\Delta_{//},T) - F(\Delta_{\perp},T).$$

The calculated coupling energy (≤ 200 K) with parameters listed in Table S1 is displayed in Fig. 4(f) in the main text, which semi-quantitatively agrees with the experimental curves.

We finally explain the conversion relationship between the coupling energy E_c and the coupling field $\mu_0 H_{Coupling}$. When E_c is higher than the Zeeman energy, the antiferromagnetic junction would be driven from the spin-flop state ($n \perp H$ for both Fe_2O_3) towards the perpendicular state. The Zeeman energy mainly arises from the net magnetization induced by DMI[S1]. Taking the exchange field of 900 T, DMI effective field of 2 T, 18 moments in each sublattice in the 4nm thickness, and 5 μ_B/atom for Fe_2O_3[S2,S3,S6], E_c is estimated to be 0.01 meV (per unit cell) for the coupling field 0.86 T.

We next compare this energy with Eq. (3) in the main text. Since the Néel temperature of Fe_2O_3 (956 K)[S14] is approximately three times of that of Cr_2O_3 (307 K)[S15], we use $A_{Fe} \approx 3\,A_{Cr}$ in the estimation. Considering $L \approx t$, $t_t = 18\,a$, where a is the monolayer distance, and the exchange coefficient J = 8 meV for the Cr_2O_3[S16], $A_{Cr} \approx Ja$, we get E_c ~ 0.049 meV (per unit cell), in the same order compared with the estimated value from the experiment. The relatively smaller experimental value could be due to the relaxation in the Fe_2O_3 in the out-of-plane direction, and the fact that the interfacial Fe and Cr moments are not identically parallel or antiparallel[S17].

Table S1. parameters for coupling energy calculation

t (nm)	$\Delta_{//}$ (10^{-2}meV)	$\Delta_{\perp}$ (10^{-2}meV)	λ (10^{-4}meV/K)	b (10^{-3}meV)
3.0	2.50	1.50	1.67	8.33
3.5	1.98	1.19		
4.1	3.44	2.82		14.17
4.4	2.99	2.45		

Supplementary References

S1. Dzyaloshinsky, I. A thermodynamic theory of "weak" ferromagnetism of antiferromagnetics. *J. Phys. Chem. Solids* **4**, 241–255 (1957).

S2. Williamson, S. J. & Foner, S. Antiferromagnetic Resonance in Systems with Dzyaloshinsky-Moriya Coupling; Orientation Dependence in α-Fe_2O_3. *Phys. Rev.* **136**, A1102–A1106 (1964).

S3. Sulymenko, O. R. et al. Terahertz-Frequency Spin Hall Auto-oscillator Based on a Canted Antiferromagnet. *Phys. Rev. Appl.* **8**, 064007 (2017).

S4. Lebrun, R. et al. Long-distance spin-transport across the Morin phase transition up to room temperature in ultra-low damping single crystals of the antiferromagnet α-Fe_2O_3. *Nat. Commun.* **11**, 6332 (2020).

S5. Elliston, P. R., & Troup, G. J. Some antiferromagnetic resonance measurements in α-Fe_2O_3. *J. Phys. C. Solid State Phys.* **1**, 169 (1968).

S6. Han, J. et al. Birefringence-like spin transport via linearly polarized antiferromagnetic magnons. *Nat. Nanotechnol.* **15**, 563–568 (2020).

S7. Nozaki, T. & Sahashi, M. Magnetoelectric manipulation and enhanced operating temperature in antiferromagnetic Cr_2O_3 thin film. *Jpn. J. Appl. Phys.* **57**, 0902A2 (2018).

S8. Cheng, Y. et al. Anisotropic magnetoresistance and nontrivial spin Hall magnetoresistance in Pt/α−Fe_2O_3 bilayers. *Phys. Rev. B* **100**, 220408 (2019).

S9. Fischer, J. et al. Large spin Hall magnetoresistance in antiferromagnetic α-Fe_2O_3/Pt Heterostructures. *Phys. Rev. Appl.* **13**, 014019 (2020).

S10. Ross, A. et al. Structural sensitivity of the spin Hall magnetoresistance in antiferromagnetic thin films. *Phys. Rev. B* **102**, 094415 (2020).

S11. Kuiper, P., Searle, B. G., Rudolf, P., Tjeng, L. H. & Chen, C. T. X-ray magnetic dichroism of antiferromagnet Fe_2O_3: The orientation of magnetic moments observed by Fe 2p x-ray absorption spectroscopy. *Phys. Rev. Lett.* **70**, 1549–1552 (1993).

S12. Gota, S., Gautier-Soyer, M. & Sacchi, M. Magnetic properties of Fe_2O_3 (0001) thin layers studied by soft x-ray linear dichroism. *Phys. Rev. B* **64**, 224407 (2001).

S13. Liu, Z.Y. & Adenwalla, S. Oscillatory interlayer exchange coupling and its temperature dependence in $[Pt/Co]_3$/NiO/$[Co/Pt]_3$ multilayers with perpendicular anisotropy. *Phys. Rev. Lett.* **91**, 037207 (2003).

S14. Díaz-Guerra, C., Pérez, L., Piqueras, J. & Chioncel, M. F. Magnetic transitions in α-Fe_2O_3 nanowires. *J. Appl. Phys.* **106**, 104302 (2009).

S15. Qiu, Z. et al. Spin colossal magnetoresistance in an antiferromagnetic insulator. *Nat. Mater.* **17**, 577–580 (2018).

S16. Samuelsen, E. J., Hutchings, M. T., & Shirane, G. Inelastic neutron scattering investigation of spin waves and magnetic interactions in Cr_2O_3. *Physica* **48**, 13–42 (1970).

S17. Mauri, D., Siegmann, H. C., Bagus, P. S. & Kay, E. Simple model for thin ferromagnetic films exchange coupled to an antiferromagnetic substrate. *J. Appl. Phys.* **62**, 3047 (1987).
